# Low-Temperature Plasma-Activated Medium Inhibited Proliferation and Progression of Lung Cancer by Targeting the PI3K/Akt and MAPK Pathways

**DOI:** 10.1155/2022/9014501

**Published:** 2022-03-18

**Authors:** Ying Li, Yang Lv, Yu Zhu, Xiaodong Yang, Boya Lin, Mengqing Li, Yaqi Zhou, Zhibo Tan, Eun Ha Choi, Junjie Wang, Shubin Wang, Yajie Liu

**Affiliations:** ^1^Department of Radiation Oncology, Shenzhen Key Laboratory of Gastrointestinal Cancer Translational Research, Cancer Institute, Peking University Shenzhen Hospital, Shenzhen Peking University-Hong Kong University of Science & Technology Medical Center, Shenzhen 518036, China; ^2^State Key Laboratory of Advanced Electromagnetic Engineering and Technology, Huazhong University of Science and Technology, Wuhan 430030, China; ^3^Department of Oncology, Shenzhen Key Laboratory of Gastrointestinal Cancer Translational Research, Cancer Institute, Peking University Shenzhen Hospital, Shenzhen 518036, China; ^4^Accurate International Biotechnology (GZ) Co. Ltd., Guangzhou 510700, China; ^5^Department of Otorhinolaryngology, Peking University Shenzhen Hospital, Shenzhen 518036, China; ^6^Department of Radiation Oncology, Peking University Shenzhen Hospital, Shenzhen 518036, China; ^7^Department of Electrical and Biological Physics, Plasma Bioscience Research Center, Applied Plasma Medicine Center, Kwangwoon University, Seoul 01897, Republic of Korea; ^8^Department of Radiation Oncology, Peking University Third Hospital, Beijing 100083, China

## Abstract

Low-temperature plasma, an engineered technology to generate various reactive species, is actively studied in cancer treatment in recent years, yet mainly by using a traditional 2D cell culture system. In this study, we explored the effect of the plasma-activated medium (PAM) on lung cancer cells *in vitro* and *in vivo* by using a 3D cell culture model. The results showed that PAM markedly inhibited 3D spheroid formation and downregulated stemness-related gene expression. We found that reactive oxygen species (ROS) penetrated throughout the whole spheroids and induced cell death surrounding and in the core of the tumor spheroid. Besides, PAM treatment suppressed migration and invasion of lung cancer cells and downregulated epithelial-mesenchymal transition- (EMT-) related gene expression. In the mouse xenograft model, the tumor volume was significantly smaller in the PAM-treated group compared with the control group. By using transcriptome sequencing, we found that PI3K/Akt and MAPK pathways were involved in the inhibition effects of PAM on lung cancer cells. Therefore, our results indicated that PAM exhibits potential anticancer effects on lung cancer and provides insight into further exploration of PAM-induced cell death and translational preclinical use.

## 1. Introduction

In the last decade, low-temperature plasma technology and devices have significantly advanced our understanding of the inhibition effects on cancer cells and as promising tools for the cancer treatment [1, 2]. Plasma can affect cancer cells and tissues either directly from the plasma discharge or via exposure to cell culture medium treated by plasma processes beforehand [3]. Plasma-activated medium (PAM) is recognized as an easier application, relatively stable, and lower cost and can be applied to a distant solid tumor, thus presenting a significant advantage in antitumor applications. Plasma interacts with nitrogen, oxygen, and water molecules during discharging and generates short-lived reactive species such as singlet oxygen (O^1^), hydroxyl radicals (^•^OH), nitric oxide (NO^•^), superoxide anion (O_2_^•−^). These short-lived ROS/RNS then might recombine or react with organic molecules and water when plasma plume bombarded onto the treated medium, consequently, remaining hydrogen peroxide (H_2_O_2_), nitrite (NO_2_^−^), and nitrate (NO_3_^−^) as major long-lived reactive species in PAM [4], making the PAM “activated” and gain the potential to kill cancer cells.

In PAM, the long-lived species, especially H_2_O_2_, are mainly responsible for genotoxic and cytotoxic effects on cancer cells [5]. Kurake et al. [6] reported that there is a synergistic effect that NO_2_^−^ enhances the H_2_O_2_ killing ability, indicating that long-lived species (especially H_2_O_2_) play a major role in PAM killing effects; however, they do not account for the whole killing effects of PAM. Currently, PAM should be used with attention to its storage conditions. The reactions between the reactive species and some specific amino acids in the culture medium, especially cysteine and methionine, are mainly responsible for the degradation. The long-lived species in PAM are stable for several days to 1 month at −80°C, and PAM loses its antitumor effect in about 18 h if stored at room temperature, indicating that the higher the storage temperature, the faster the degradation. Therefore, to stabilize the reactive species, PAM should be stored under a low temperature (−80 or −150°C) [7].

Lung cancer is the most common cause of cancer-related mortality worldwide. Although our understanding of treatment options for lung cancer has improved, it is still the leading cause of cancer deaths, with a 10-year survival rate of less than 7% after diagnosis in all stages of the lung cancer [8]. Lung cancer cells proliferating in a three-dimensional (3D) culture system can better mimic the structural complexity, genetic signature, and physiological functionality of *in vivo* tumors than traditional 2D cultures [9, 10]. 3D cultures enable cancer cells to overexpress “stemness” genes and enrich the formation of stem-like cancer cell subpopulations, providing new insights into cancer treatment and cancer stem cell research [11]. The demand for the 3D cell culture system on anticancer drug screening and discovery makes it one of the fastest-growing experimental approaches in life sciences [12]. 3D cultures for assessing the anticancer effects of plasma technology are also increasing in recent years [7, 13, 14]. However, methodologically speaking, the 3D spheroids were generated by the centrifugation method in most studies of PAM anticancer effects and it is reported that the basement membrane extract- (BME-) based *in vitro* 3D culture method is more feasible to stimulate the solid tumor growth pattern *in vivo* than conventional 3D suspension culture [15]. We had previously established that BME-based 3D culture enriches and amplifies stemness gene expression of cancer cells, demonstrating that *in vitro* 3D culture is a good model for studying cancer stem-like cells [16]. However, the cellular signal pathway in 3D spheroids after PAM treatment is beyond understanding. The studies of PAM antitumor effects are mainly performed in the 2D cell culture system and neglect the defect of cell-cell and cell-matrix interactions, while using a 3D cell culture system can remedy the situation and provide a better evaluation system for the antitumor effect of PAM.

The phosphatidylinositol-3-kinase (PI3K)/Akt pathway plays a crucial role in both tumorigenesis and the progression of the lung cancer [17]. Pieces of evidence show that PAM normally suppresses the PI3K/Akt pathway, thus inhibiting tumor growth. Kim et al. clarified that plasma inhibited tumor progression by reducing phospho-Akt levels in the head and neck cancer [18]. The inhibited proliferation of glioma cells by PAM treatment was due to downregulating the survival signaling molecule, Akt kinase, in the brain tumor field [19]. Downregulation of the survival and proliferation signaling networks is consistent with the results that PAM downregulated phospho-Akt. On the other hand, the mitogen-activated protein kinases (MAPKs) are important modulators of cellular responses, especially under stress (e.g., oxidation) [20, 21]. Various studies have verified that direct plasma treatment or PAM induced cancer cell apoptosis by a mechanism of the activation of MAPKs, including c-JUN N-terminal kinase (JNK), extracellular-regulated kinase (ERK), and p38 [22, 23]. However, in the 3D culture system, the regulation of tumor spheroid signaling pathways by PAM treatment has not been well studied in 3D culture systems.

Here, we used a BME-based *in vitro* 3D culture method and established *in vivo* models to evaluate the inhibitory effect of PAM on lung cancer cell growth and migration, as well as the signal pathways of 3D tumor spheroids after PAM treatment. Our results show that PAM has significant antineoplastic potential on lung cancer cells cultured in 3D *in vitro* and xenografts *in vivo*. This is achieved by inhibiting the PI3K/Akt pathway and activating MAPK pathways, indicating that PAM treatment could be advantageous for the improvement of clinical outcomes for lung cancer.

## 2. Materials and Methods

### 2.1. 2D and 3D Cell Culture

A549, SK-MES-1, H23, and H520 cells (American Type Culture Collection) were maintained in high-glucose Dulbecco's modified Eagle medium (DMEM, Life Technologies) containing 10% fetal bovine serum (Gibco) and 1% streptomycin and penicillin and maintained in a humidified atmosphere of 5% CO_2_ at 37°C. For 2D cell culture, cells were seeded on the well plate at a demanding cell density directly with DMEM culture medium.

For the generation of 3D spheroids, we used a basement membrane extract- (BME-) based method [15]. Briefly, 50 *μ*l of Matrigel (Corning) was used to coat the 96-well plate and form a reconstituted basement membrane at 37°C for 1 h. Then, cell suspension with a density of 1 × 10^4^ cells/well was seeded on the top of the Matrigel. After spheroid formation at day 4, those spheroids were then used for subsequential experiments.

### 2.2. Cell Viability Assay

The proliferative ability was detected using Cell Counting Kit-8 (CCK8) (Beyotime, China) proliferation assay according to the manufacture's protocol. Briefly, cells from the 2D culture condition were seeded at a density of 3 × 10^3^ in a 96-well flat-bottom plate and cultured overnight. Cells were treated with 100 *μ*l of PAM (activated with 1, 5, 10, 15, and 20 min by plasma jet) and incubated for 72 h or treated with 100 *μ*l of PAM (activated with 20 min) and incubated for 24, 48, and 72 h. The ROS scavenger N-acetyl-L-cysteine (NAC) (20 *μ*M, Sigma Aldrich) and catalase (0.6 mg/ml, Beyotime Institute of Biotechnology, China) and RNS scavenger, 2- to 4-carboxyphenyl-4,4, 5,5-tetramethylimidazoline-1-oxyl-3-oxide (cPITO, 100 *μ*M, Beyotime Institute of Biotechnology, China) were used to treat cells 1 h pre-PAM treatment. Cell viability was detected by using CCK8, and the absorbance reading at 450 nm was taken by a microplate reader (Multiskan GO, Thermo Scientific). The cell number was counted after 24 h PAM treatment (activated with 20 min) using Trypan Blue under an automated cell counter (Bio-Rad, TC-20). Nontreated cells were used as a control group. All experiments were repeated at least 3 times.

### 2.3. Live/Dead Assay

To visually observe the killing effect of PAM, the lung cancer cells were seeded onto a 24-well plate with a cell density of 5 × 10^4^ cells/well and incubated overnight. The cells were then treated with or without PAM for 48 h. Then, the cells were washed with phosphate-buffered saline (PBS), followed by staining with calcein-AM/PI double-staining kit (Yeasen, Shanghai, China) following the manufacturer's instructions. Briefly, 2 *μ*M of calcein-AM and 4.5 *μ*M of PI were added to the cells and incubated for 15 min at 37°C, followed by observation under a fluorescence microscope (Leica, Germany). The relative intensity of PI-indicating dead cells was analyzed using ImageJ software of images in five independent experiments.

For 3D spheroids, after spheroid formation (at day 4), the culture medium was changed to PAM (20 min activated) and treated for 48 h. Then, a similar staining process was performed using calcein-AM and PI. The images were taken using a fluorescence microscope (Leica, Germany). The relative intensity of PI-indicating dead cells was analyzed using ImageJ software.

### 2.4. Cell Morphology

Lung adenocarcinoma (A549 and H23) and lung squamous carcinoma (SK-MES-1 and H520) cells were cultured in 2D and 3D systems. The images of 2D cells and 3D spheroids (at day 6 after seeding) were taken using microscopy (Leica, Germany). The average diameters of 3D spheroids on day 6 were measured and presented as mean ± standard deviation.

### 2.5. pH Value Detection

To characterize the pH value of PAM before using it to cells, the complete DMEM medium was treated with a plasma jet for 1, 5, 10, and 20 min, respectively. Immediately after the PAM was prepared, the pH value was measured using a pH meter (Mettler Toledo equipment). Nontreat DMEM medium, deionization (DI) water, and PBS were used as controls. Each group was measured using at least 3 separate samples, and the average pH values were used to make the bar graph.

### 2.6. Measurement of H_2_O_2_ and NO_x_ in the PAM

To detect the H_2_O_2_ and NO_x_ levels with different plasma-activating times of PAM, we first treated the complete DEME medium with a plasma jet of 1, 5, 10, 15, and 20 min and a nontreated medium was used as blank. Immediately after PAM was prepared, the H_2_O_2_ level was measured according to the instructions of the QuantiChrom Peroxide Assay Kit (BioAssay System, CA, USA) and the NO_x_ level was measured following the instructions of the nitric oxide assay kit (BioAssay System, CA, USA).

### 2.7. Quantitative Real-Time PCR

To extract RNA from lung cancer spheroids, after spheroid formation (at day 4), the culture medium was changed to PAM (20 min activated), and treated for another 48 h, nontreated spheroids were used as the control group. Then, those spheroids were collected for total RNA extraction using the RNA extraction kit (TaKaRa Biomedical Technology, Japan) according to the manufacturer's protocol. Complementary DNA was synthesized using an equivalent amount of total RNA (1 *μ*g) in a 20 *μ*l reverse transcriptase reaction mixture using a cDNA synthesis kit (TaKaRa Biomedical Technology, Japan). The cDNA was subsequently served as the template for qRT-PCR and detected using TB Green Premix Ex Taq II (TaKaRa Biomedical Technology, Japan). The amplification was performed using the following protocol: 40 cycles of 95°C for 10s, 60°C for the 20s, and 72°C for 30s. 18S was used as the reference gene for normalization. The primers used in this study were designed and purchased from a company (Sangon, Shanghai, China). Primers are listed in Table 1 for detailed information.

### 2.8. Cell Migration Assay

Both 2D- and 3D-cultured lung cancer cells were seeded into 6-well plates for 24 h to reach 85~90% confluency. The cell monolayer was manually scratched in a straight line with a 1 ml pipette tip and washed with the DMEM medium to remove cells detached from the plates. The scratches were imaged by a microscope (Leica, Germany) at 0 h. The cells were then cultured in the DMEM medium (control group) and 20 min plasma jet-activated DMEM medium (PAM group). After 24 h, the cells were fixed using 4% paraformaldehyde solution and stained with crystal violet (Sigma-Aldrich, USA), and then, gap images were taken using a microscope. The relative cell migration rates of 2D- and 3D-cultured A549 were then calculated using Image J software of the images in five independent experiments.

### 2.9. Transwell Invasion Assay

We performed the invasion assay in transwell chambers (Corning, NY, USA) with inserts that were precoated with Matrigel (BD Biosciences, Franklin, USA). Briefly, 100 *μ*l of Matrigel (50 mg/l, 1 : 8 diluted) was coated on the surface of the upper chamber and air dried at room temperature. 2D- and 3D-cultured lung cancer cells were resuspended in a serum-free DMEM medium. The cell density was adjusted to 8 × 10^4^/ml, and 200 *μ*l was added to the upper chamber. The bottom chambers were filled with 500 *μ*l of culture medium (for the control group) or PAM (20 min activated, for the PAM group). After incubation for 24 h, the upper chamber of transwell was removed and the cells were washed with PBS. Those unmigrated cells on the inner surface were gently wiped off using a cotton swab. Cells on the inner membrane were fixed in 4% paraformaldehyde for 20 min, stained with crystal violet, and counted as an invaded cell number using ImageJ software of the representative images in five independent experiments.

### 2.10. Western Blot

To extract the protein, A549 spheroids were treated with PAM for 48 h and nontreated spheroids were used as control. Then, the Matrigel was removed using a cell recovery solution (Corning, USA) and the spheroids of each group were harvested and lysed in RIPA buffer (Sangon Biotech Co. Ltd.) for protein extraction. The protein concentration of lysates was measured using a BCA Protein Assay Kit (Sangon Biotech Co. Ltd.). Next, 20 *μ*g protein per lane was electrophoretically separated using 4–15% sodium dodecyl sulfate-polyacrylamide gel electrophoresis (SDS-PAGE) (Bio-Rad) and then was transferred to PVDF membranes (Millipore, MA, USA). The membranes were blocked using 5% bovine serum albumin (BSA) for 1 h at room temperature and then incubated with primary antibodies specific for PI3K, p-PI3K, Akt, p-Akt, ERK, p-ERK, p38, p-p38, JNK, p-JNK, and *β*-actin (all from Cell Signaling Technology, MA, USA). Subsequently, horseradish peroxidase- (HRP-) conjugated secondary antibodies of goat anti-rabbit and goat anti-mouse (AbD Serotec, USA) were used to detect the corresponding primary antibody. Immunoblots were visualized using an enhanced chemiluminescence (Beyotime Institute of Biotechnology, China).

### 2.11. Xenograft Tumor Model

Animal experiments were performed in accordance with the animal research committee guidelines in Peking University Shenzhen Hospital. Individually, we used 2D- and 3D-cultured cells to establish the mice xenografts. For 2D-cultured cells, a total of 1 × 10^7^ cells were suspended in 50 *μ*l of DMEM medium and 50 *μ*l Matrigel growth factor reduced (Corning), or for the 3D culture, A549 spheroids were mixed with 100 *μ*l Matrigel and implanted subcutaneously into the flank of six-week-old female Balb/c nude mice (GemPharmatech Company, Guangdong, China). After the tumor formation, the mice were divided into a control group (*n* = 4) and a PAM-treated group (*n* = 4). For the control group, mice received 100 *μ*l of DMEM medium, whereas in the PAM-treated group received 100 *μ*l of PAM (20 min activated) by subcutaneous injection. The subcutaneous administration was performed continuously for 5 days a week, for 2 weeks. To evaluate the antitumor effects, the tumor volume was calculated using the formula: 1/2 × (largest diameter) × (smallest diameter)^2^ as described in a previous study [24]. On day 22, the mice were sacrificed and the tumors were harvested and weighed.

### 2.12. Hematoxylin & Eosin Staining and Immunohistochemical Determination of Ki67

Hematoxylin & eosin (H&E) staining were performed on xenograft of the control and PAM-treated groups. We used two tumor sections from each mouse to make slides, and 5 fields were randomly selected from each slide, with a total of more than 10 fields in each group study. All images were taken under a Leica microscope (Wetzel Gmbh, Germany). An anti-Ki67 monoclonal antibody (ab16667, Abcam) was used to detect the proliferation of tumor cells in tissue slides of the control and PAM-treated groups. To quantify Ki67 expression, the Ki67-positive cells were calculated using ImageJ software.

### 2.13. Detection for Transcriptome Sequencing and KEGG Enrichment Analysis

Total RNA of PAM-treated group and control group A549 3D spheroids was extracted using TRIzol (Invitrogen Life Technologies, USA). The purity of RNA was checked by a NanoPhotometer Spectrophotometer (IMPLEN, USA). 1 *μ*g RNA of each sample was used as input material for the RNA sample preparations to synthesize cDNA. The NEBNExt Ultra™ RNA Library Prep Kit for Illumina^®^ (NEB, Ipswich, MA, USA) was used to generate sequencing libraries following the manufacturer's recommendations for each sample. TreSeq PE Cluster Kit v3-cBot-HS (Illumina) was used to perform the cluster of the index-coded samples. After cluster generation, the library was sequenced on a platform of Illumina NovaSeq (Tianjin, China). To understand molecular interaction and signaling pathways, the ClusterProfiler R package (with a corrected *P* value < 0.05) was used to test the statistical enrichment of differentially expression genes (DEGs) in KEGG pathways.

### 2.14. Statistical Analysis

Each experiment involved was repeated at least three times independently, and the data was represented by the means ± standard deviation of replicate sets. GraphPad Prism version 7.00 (GraphPad Software, USA) was used to analyze the data. Student's *t*-test was used for evaluating significance between groups. The statistically significant differences were based on ^∗^*P* < 0.05, ^∗∗^*P* < 0.01, ^∗∗∗^*P* < 0.001, and ^∗∗∗∗^*P* < 0.0001.

## 3. Results

### 3.1. Plasma Jet Device

**Figure 1:**
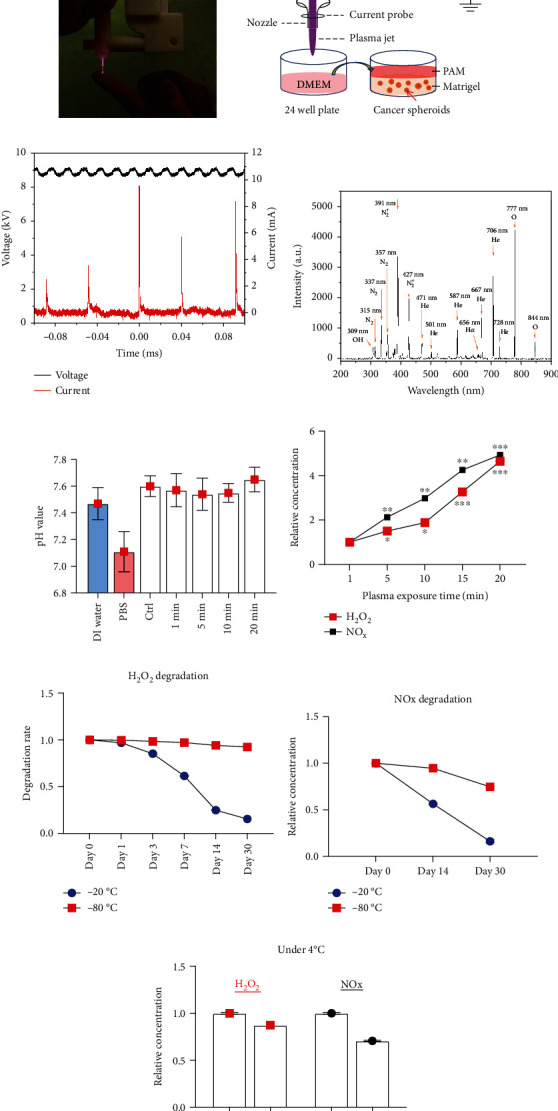
(a) Image of the hand-held portable low-temperature plasma jet. (b) Schematic of helium plasma jet for the preparation of PAM. (c) Typical voltage and current waveforms of the helium plasma jet at a discharge power of 1.05 W and voltage of 9 kV. (d) OES measurement of plasma jet discharging with He gas and O_2_ gas with a wavelength ranging from 200 to 900 nm. (e) pH value of DI water, PBS, and different exposure times of plasma-activated DMEM medium. (f) The relative level of H_2_O_2_ and NO_x_ in different exposure times of PAM. (g) Degradation of H_2_O_2_ under the storage at **−**20 and **−**80°C. (h) Degradation of NO_x_ under storage at **−**20 and **−**80°C. (i) Degradation of H_2_O_2_ and NO_x_ under storage at 4°C.

A hand-held portable type of a helium plasma jet (Figure 1(a)) was used to activate the DMEM cell culture medium in this study.  Figure 1(b) shows the schematic of the helium plasma jet. It is driven by a DC power supply at a voltage of 4.7 kV. A needle with a radius of 50 *μ*m connects with the power supply through a resistor ballast of 500 M*Ω* in series. The needle gets out of a nozzle with a diameter of 1.5 mm. The plasma discharge was performed using a mixed working gas of helium at a flow rate of 2 liters per minute and oxygen at a flow rate of 10 milliliters per minute. A plasma plume is generated at the tip of the needle with around 3 cm in length. A 24-well plate with a 1.2 ml DMEM cell culture medium was placed under the plasma plume, and the distance between medium surfaces to the nozzle is around 1 cm.  Figure 1(c) shows the waveforms of the applied voltage of 9 kV and the current for the plasma discharge. The optical emission spectroscopy (OES) measured by a spectrometer (HR4000, Ocean Optics, FL) was used to identify the ROS and RNS generated by the helium plasma jet. The spectrum was measured at a wavelength ranging from 200 to 900 nm. The OES showed that discharge using He and O_2_ gas mixtures achieved OH (309 nm), O777 (777 nm), O844 (844 nm), H*α* (656 nm) peaks, and N_2_ emission from the second positive system (300~440 nm, with the dominant peak at 391 nm) (Figure 1(d)), which are considered to be the important ROS and RNS of PAM.

As shown in Figure 1(e), the pH value of different exposure times of PAM ranged from 7.5 to 7.8, which has no significant difference with complete DMEM medium (pH 7.6), DI water (pH 7.5), and PBS (pH 7.1). The relative H_2_O_2_ level and NO_x_ level showed a plasma-activating time-dependent manner (Figure 1(f)). Since degradation of long-lived species in PAM is currently an important issue, we performed kinetic analyses of H_2_O_2_ and NO_x_ degradation under storage at different temperatures. We found that the H_2_O_2_ level can sustain up to 1 month when stored at −80°C; however, it degraded by about 38% on day 7 and 75% on day 14 when stored at −20°C (Figure 1(g)). The NO_x_ level degrades faster regardless of −20 or −80°C storage (Figure 1(h)). However, both H_2_O_2_ and NO_x_ levels gradually degraded after 4 h storage at 4°C (Figure 1(i)). These findings indicate that storage in −80°C can inhibit PAM degradation over long periods.

### 3.2. Cytotoxicity of PAM in 2D Lung Cancer Cells

**Figure 2:**
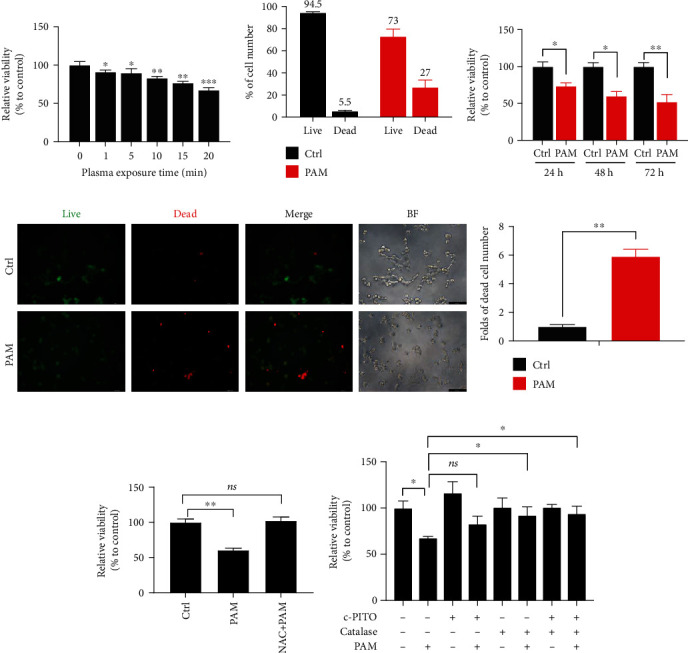
Anticancer effect of PAM on 2D lung cancer cells. (a) Cell viability according to different activated times of PAM on SK-MES-1 cells. (b) Cell counting of live and dead A549 cells using trypan blue dye of control and PAM-treated groups. (c) Cell viability according to different incubation times of 24, 48, and 72 h. (d) Live/dead assay after PAM treatment of SK-MES-1 cells; nontreated cells were used as the control group. (e) The qualification of live and dead cell intensity using ImageJ software. 2D cell viability inhibition was attenuated by (f) ROS scavenger NAC and (g) H_2_O_2_ scavenger catalase and RNS scavenger, cPITO. ^∗^*P* < 0.05, ^∗∗^*P* < 0.01, and ^∗∗∗^*P* < 0.001.

Since the anticancer effects of PAM are well described in 2D culture-based *in vitro* studies [16, 25], we initially determined the cytotoxicity of PAM using CCK8 assay and cell counting on 2D-cultured lung cancer cells, laying the foundation for the PAM effects on 3D cell spheroids. PAM showed a dose-dependent antiproliferation as the plasma exposure time of PAM on 2D cancer cells (Figure 2(a)) and a more decrease of the cell number in the PAM-treated group (Figure 2(b)). Extended incubation time to 72 h revealed that the PAM effect on cell viability is a continuous and irreversible process (Figure 2(c)). The live/dead assay further showed that PAM induced 5.9-fold of cell death as compared with that of the control group (Figures 2(d) and 2(e)), proving the anticancer potential of PAM on lung cancer cells.

To further investigate the mechanism of specific ROS/RNS that plays a major role in antitumor effect, A549 cells were treated with the ROS scavenger NAC, the H_2_O_2_ scavenger catalase, and the NO^•^ scavenger cPITO 1 h before PAM treatment. The results showed that pretreatment with NAC completely reversed the PAM effect (Figure 2(f)). In contrast, cPITO partially reversed the PAM effect, although with no statistical significance compared with that of the control group, while the addition of catalase alone or combined with cPITO showed a more inhibitory effect on cell viability than cPITO (Figure 2(g)). These findings suggested that the antitumor effect of PAM on cell viability is mainly exerted by ROS, especially H_2_O_2_, but NO^•^ and its derivatives also have a synergic effect on H_2_O_2_, which is consistent with the previous study [6]. Since we aimed to study the PAM effects on 3D lung cancer spheroids, Zhang et al. [13] reported that PAM induced less toxic effects on 3D tumor spheroids than 2D monolayer cells due to the spatial organization of 3D spheroids. Thus, we used PAM activated by 20 min plasma treatment for the following 3D-based studies.

### 3.3. PAM-Induced ROS Accumulation in 3D Tumor Spheroids

**Figure 3:**
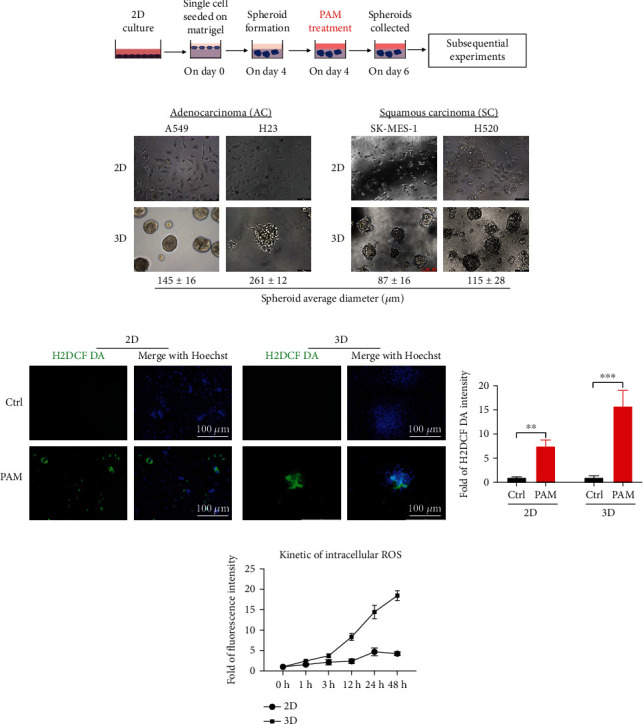
(a) Schematic representation of the experiment workflow. (b) Bright field images of 2D and 3D culture lung adenocarcinoma cells (A549 and H23) and lung squamous carcinoma cells (SK-MES-1 and H520) at day 6; scale bar, 100 *μ*m. (c) ROS accumulation in PAM-treated 2D cells and 3D spheroids; scale bar, 100 *μ*m. Nontreated cells and spheroids as control. (d) Quantification of the relative intracellular ROS level in 2D cells and 3D spheroids. (e) Kinetic analysis of the intracellular ROS level in 2D cells and 3D spheroids measured at different time intervals following PAM treatment.

Figure 3(a) presented the schematic of the 3D cell culture and experiment workflow. We cultured two lung adenocarcinoma cell lines (A549 and H23) and two lung squamous carcinoma cell lines (SK-MES-1 and H520) in the 3D system to show the morphology of lung cancer multicellular spheroids (Figure 3(b)), and 2D cell morphology of each cell line was also presented. All cell lines have a similar sphere formation time at around 4 days with diameters from 71 to 273 *μ*m. Though the spheroids in Matrigel presented in various sizes, studies showed that long time cultivation in a 3D Matrigel culture system strongly increased cell metabolic competence [26].

PAM-induced intracellular ROS accumulation has been well studied in 2D-cultured cancer cells [27], and ROS penetration in 3D spheroids was also observed in the conventional suspension 3D culture system [5]. To confirm PAM-induced ROS accumulation in BME-based 3D spheroids, firstly, we detected intracellular ROS in 2D cells and 3D spheroids after PAM treatment for 48 h. Figures 3(c) and 3(d) showed that ROS accumulated in 2D cells (7.5-fold of nontreated cells) and the penetration and diffusion of ROS throughout the 3D spheroids, even at the center, were observed (15.8-fold of nontreated spheroids). This is consistent with previous findings that Zhang et al. [13] also reported that liquid coverage enabled the ROS to penetrate inside the 3D tumor spheroids. We incubated spheroids with PAM for 48 h (presented a long-time incubation); therefore, the ROS could penetrate and distribute throughout the spheroids.

Interestingly, by kinetic analysis of the intracellular level, we found that ROS accumulation increased after 1 h PAM incubation in 2D cells and 3D spheroids. In the 2D culture, the ROS level peaked at 24 h and then dropped a little at 48 h, whereas in the 3D culture system, it started to rise after 3 h and was still on uptrend until 48 h (Figure 3(e)). This may suggest that after PAM treatment, the ROS can be captured in 3D spheroids and restrained for a longer time.

### 3.4. PAM Inhibited Tumorigenesis in 3D-Cultured Lung Cancer Cells

**Figure 4:**
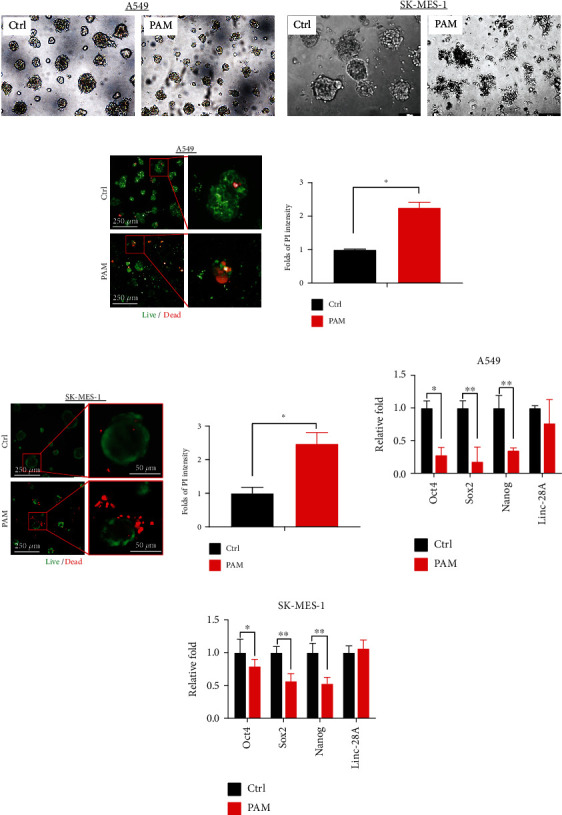
PAM inhibition effect in 3D lung cancer spheroids. Morphological change of 3D A549 spheroids (a) and 3D SK-MES-1 spheroids (b) after PAM treatment. Live/dead assay of 3D A549 spheroids (c) and 3D SK-MES-1 spheroids (d) with PAM treatment for 48 h; the qualification of dead cells by ImageJ software were presented in the right panel. Stemness-related mRNA expression of (e) 3D A549 spheroids and (f) 3D SK-MES-1 spheroids. Nontreated spheroids were used as control. Significance was presented as ^∗^*P* < 0.05 and ^∗∗^*P* < 0.01 in all experiments.

To further determine the effect of penetrated ROS on 3D tumor spheroids, sphere morphology, live/dead assay, and stemness-related mRNA detections were performed. Figures 4(a) and 4(b) showed PAM treatment-induced distinct morphological changes of A549 and SK-MES-1 3D spheroids and inhibited tumor spheroid formation as shown with the smaller sphere size and irregular sphere shape.

To verify the anticancer effect of PAM, we performed a live/dead assay on 3D spheroids. PAM treatment induced significant cell death as 2.2 for A549 spheroids (Figure 4(c)) and 2.5-fold for SK-MES-1 spheroids (Figure 4(d)) than that of the relative control group. The dead cells were presented around the tumor spheroids and in the core of those spheroids. According to the ROS distribution throughout the whole spheroids (Figure 3(c)), we inferred that ROS penetration inside spheroids induced cell death.

As PAM treatment significantly suppressed tumor spheroid growth, which can be illustrated as self-renewal of stem-like cells or tumor-initiating cells, we then collected the spheroids after PAM treatment and then analyzed the stemness-associated transcription factors. As shown in Figures 4(e) and 4(f), stemness markers including Oct4, Sox2, and Nanog were significantly downregulated in different levels in lung cancer cells after PAM treatment, indicating that PAM inhibited cancer initiation by reducing the self-renewal ability of cancer cells.

### 3.5. PAM Suppressed the Migration and Invasion of 3D-Cultured Lung Cancer Cells

**Figure 5:**
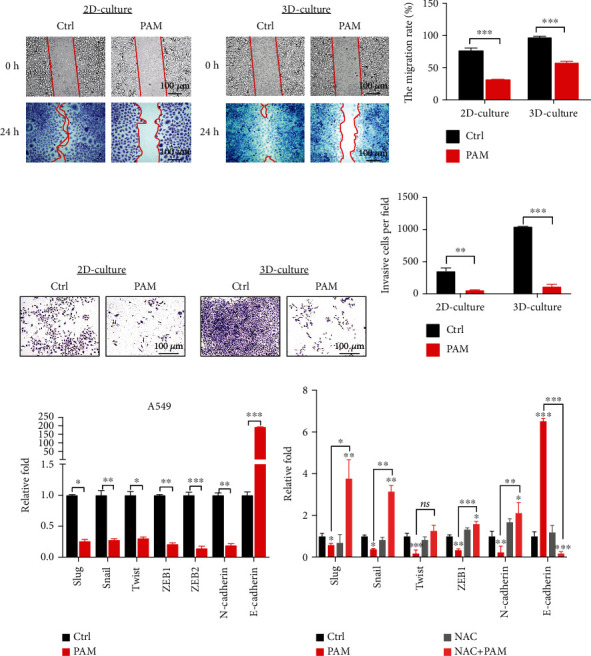
PAM inhibited lung cancer cell migration and invasion and downregulates EMT mRNA expression. (a) A migration assay was performed to evaluate the migration of A549 cells in 2D and 3D cultures after being treated with PAM. The image of 0 h and 24 h was recorded by a microscope, respectively. Scale bar, 100 *μ*m. (b) The percentage of gap area covered at 24 h for PAM and control groups was normalized to 0 h gap area of each group. Data were as means ± SD obtained from five individual experiments. (c) Invaded cells from 2D and 3D lung cancer cells imaged in a bright-field microscope. Scale bar, 100 *μ*m. (d) The number of invaded cells was quantified as in the mean ± SD of five individual experiments. (e) EMT-related mRNA expression levels in 3D-cultured A549 spheroids. (f) EMT-related markers in 3D culture A549 spheroids when treated by PAM, NAC, and NAC + PAM were shown as relative expression values, the nontreated group as control. Significance was presented as ^∗^*P* < 0.05, ^∗∗^*P* < 0.01, and ^∗∗∗^*P* < 0.001 in all experiments.

Cancer cells are characterized by the potential for metastasis and proliferation. To evaluate the effects of PAM on 3D-cultured A549, we performed migration and invasion assays on those cells in 2D and 3D cultures. PAM-treated cells underwent a decreased migration potential compared with the untreated control group, while 3D-cultured cells presented a faster migration as compared with the 2D culture cells of the control and PAM groups (Figures 5(a) and 5(b)). We then carried out experiments using transwell chambers with Matrigel. As expected, a significant decrease of invaded cell numbers in the PAM-treated group of 2D- and 3D-cultured A549 cells as compared with the control group (Figure 5(c)). In addition, in comparison with those of the 2D and 3D control groups, invaded cells have a significant increase in 3D-cultured cells (Figure 5(d)), indicating that with the 3D culture system, the cells gain more metastatic potential.

Activation of epithelial-mesenchymal transition (EMT) provides importance for understanding malignant tumor progression towards the dedifferentiated phenotype with enhanced cellular motility. Since we have observed a significant inhibitory effect of PAM on cell migration and invasion, we further examined whether PAM treatment downregulated EMT-regulating transcription factors, including Snail, Slug, Twist, ZEB family, and N-cadherin in 3D-cultured A549 cells. As shown in Figure 5(e), we found that the expression of all the above EMT-related markers was decreased by PAM treatment, while E-cadherin, an epithelial marker, was significantly increased after PAM treatment. Moreover, pretreatment of NAC significantly reversed the PAM regulation on the expression of EMT markers (Figure 5(f)). Taken together, our findings indicated that PAM significantly suppressed the cellular motility of both 2D- and 3D-cultured A549 cells.

### 3.6. PAM Inhibited Tumorigenesis of Lung Cancer *In Vivo*

**Figure 6:**
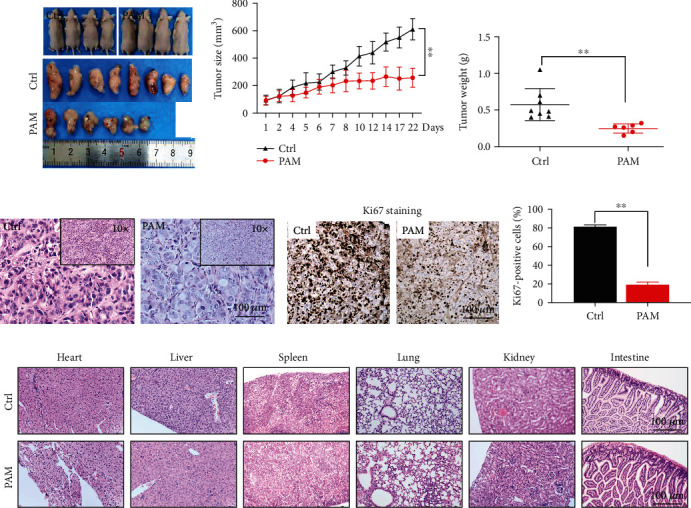
PAM inhibited lung cancer growth *in vivo*. (a) Images of mice and the xenograft with 3D spheroid in the control and PAM groups. (b) The tumor volume was assessed at the indicated time points after the onset of treatment on the established 3D xenograft. (c) The tumor weight of control and PAM group 3D xenografts. (d) H&E staining of xenograft tumor tissue in control and PAM-treated groups. Scale bar = 100 *μ*m. (e) Ki67 immunohistochemical staining of representative tumor images in control and PAM-treated groups. Scale bar = 100 *μ*m. (f) Percentage of Ki67-positive cells in the control and PAM groups. (g) Representative HE staining of the heart, liver, spleen, lung, kidney, and intestine in mice of control and PAM-treated groups. Scale bar = 100 *μ*m.

To determine the anticancer effect of PAM on lung cancer *in vivo*, we culture A549 3D spheroid and established xenograft tumors in nude mice. The images of tumor masses were observed as shown in Figure 6(a). PAM treatment significantly suppressed the growth of the tumor xenografts as compared with that of the control group (Figure 6(b)). The tumor weight was also significantly less in the PAM-treated group and around 64.2% in the control group in 3D A549 xenograft (Figure 6(c)).

H&E staining assay revealed the pathological morphology of the control and PAM groups. In the control group, tumor tissue exhibited a complete structure and regular shape of enlarged tumor cells and nuclei, while in the PAM-treated group, the tumor tissue sections were less stained and presented with an irregular shape of cells and nuclei (Figure 6(d)). The effect of PAM treatment on antitumor proliferation was determined by immunohistochemical staining of Ki67. As shown in Figure 6(e), there were fewer Ki67-positive cells in the PAM-treated group compared with the control group. The percentage of positive Ki67 cells in the PAM-treated group was significantly less (19.45 ± 2.61%) than that of the control group (81.63 ± 1.54%, *P* < 0.05) (Figure 6(f)). No significant changes in the histopathological morphology of organs were observed between the control and PAM groups throughout the experiment (Figure 6(g)).

### 3.7. Potential Pathways Related with PAM Inhibition of Lung Tumor Growth

**Figure 7:**
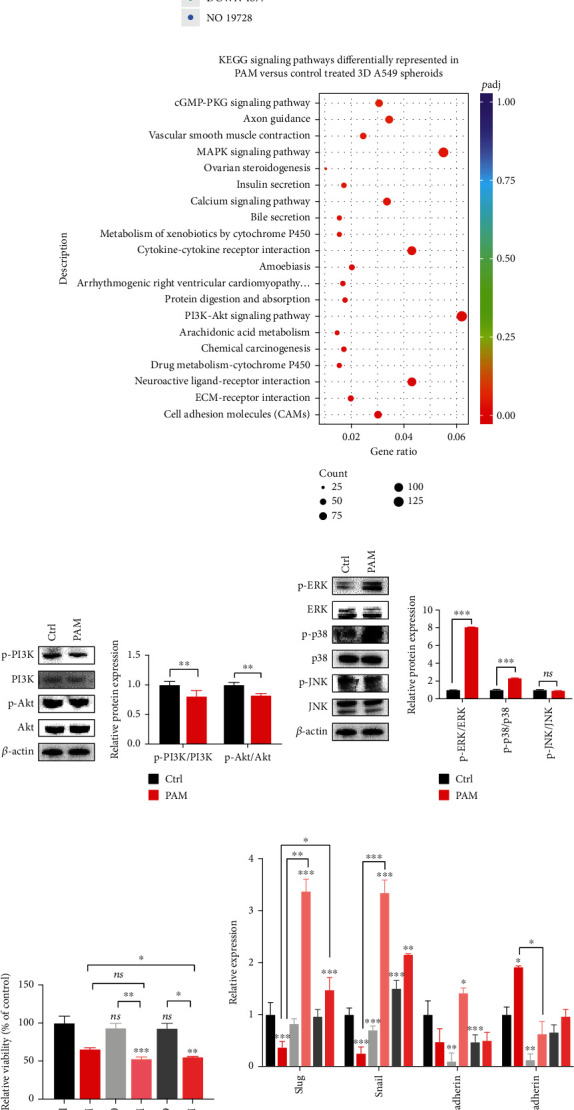
Potential pathways involved in PAM inhibition of lung tumor growth. (a) Volcano plot indicating 4319 significantly upregulated and 4877 significantly downregulated genes (*P* < 0.05; |log2 fold change | >1) in the PAM-treated group compared with the control group. (b) KEGG analysis representing signaling pathways deferentially represented in control and PAM-treated 3D A549 spheroids. (c) Left: Western blot of phosphorylated PI3K and Akt in control and PAM-treated 3D A549 spheroids. Total protein levels of PI3K and Akt were analyzed. *β*-Actin was used as a loading control. Right: quantification of phosphorylated PI3K and Akt normalized to the total amount of the same proteins. (d) Left: Western blot of phosphorylated ERK, p38, and JNK in control and PAM-treated 3D A549 cells. Total protein levels of ERK, p38, and JNK were analyzed. *β*-Actin was used as a loading control. Right: quantification of phosphorylated ERK, p38, and JNK normalized to the total amount of the same proteins. (e) The cell viability of 3D culture A549 spheroids when treated with PAM, SB203580, SB202580 + PAM, PD98059, or PD98059 + PAM was shown as relative expression values. (f)The qRT-PCR results of EMT gene markers in 3D culture A549 spheroids when treated with PAM, SB203580, SB202580 + PAM, PD98059, or PD98059 + PAM were shown as relative expression values. The results represent average plus s.d. from at least three independent experiments. ^∗^*P* < 0.05, ^∗∗^*P* < 0.01, and ^∗∗∗^*P* < 0.001; ns: not significant. (g) Schematic representation of PAM in the regulation of cell survival and migration via PI3K/Akt and MAPKs pathways in 3D-cultured lung adenocarcinoma cells.

After PAM treatment effectively inhibited lung tumor growth *in vitro* and *in vivo*, we next sought to investigate the signaling pathways during this process by using transcriptome sequencing of PAM-treated and control A549 3D spheroids. Here, 9,196 differentially expressed genes (DEGs) were found in the PAM-treated spheroids vs. the control group, including 4319 upregulated genes and 4877 downregulated genes (Figure 7(a)).

To obtain further insight into gene functions and metabolic signaling pathways, KEGG pathway enrichment analyses were performed. The two most significant enriched KEGG pathways were the PI3K/Akt signaling pathway and the mitogen-activated protein kinase (MAPK) signaling pathways in PAM-treated spheroids vs. the control group (Figure 7(b)). We then focused our attention on these two pathways.  Figure 7(c) showed that the expression levels of p-PI3K and p-Akt were decreased in the PAM-treated group than the control group. Since the PI3K/Akt signaling pathway is related to the proliferation of tumor cells, inactivation of this pathway is indicated to the inhibition of cell viability.

Mitogen-activated protein kinases (MAPKs) are important modulators of cellular responses. We analyzed the activation status of the three main subfamily proteins of MAPKs in PAM-treated 3D spheroids of A549. The results showed that the activation of the phosphorylation of ERK1/2 and p38 was increased, whereas there is no significant change of JNK protein (Figure 7(d)). Moreover, pretreatment of A549 3D spheroids with the ERK inhibitor (PD98059) and p38 inhibitor (SB203580) prior to PAM reduced cell viability more than PAM treatment alone (Figure 7(e)), suggesting a combination of antitumor effects. However, the suppression of EMT-related mRNA expression by PAM was attenuated by adding the inhibitors (Figure 7(f)), which may be due to the other pathways also involved in regulating cell migration, and the specific mechanism needs further study.

The results of the current study revealed that when PAM is applied to 3D lung cancer spheroids, it inhibits the expression of p-PI3K and p-Akt and activates the expression of p-p38 and p-ERK, which regulates the downstream signaling pathway to reduce the proliferation and migration of 3D A549 spheres (Figure 7(g)).

## 4. Discussion

Low-temperature plasma-activated solutions, such as cell culture medium, PBS, and saline, have been extensively studied and considered as candidates for the treatment of various cancers [25, 28]. The relatively long-lived H_2_O_2_ and NO derivatives are likely to play a major role in the PAM-induced cancer cell apoptosis, in which the synergic interaction of H_2_O_2_ and nitrite might generate peroxynitrite, further leading to the formation of hydroperoxide radicals, followed by apoptotic pathway activation [29, 30].

The anticancer role of PAM has been identified as inhibition of cell proliferation, migration, invasion, and cell cycle in various cancers; however, most of these studies were performed in the traditional 2D culture system. Due to more reliable prediction of the tumor environment *in vivo*, the 3D cell culture system has been developed rapidly and it is necessary to be applied to evaluate the anticancer effect of PAM.

We investigated the effects of PAM on NSCLCs using a 3D cell culture system. It inhibited the proliferation and progression of both 2D and 3D culture lung cancer cells and suppressed the spheroid formation in 3D culture by downregulating the stemness-related marker expressions. The PAM anticancer effect was also confirmed with 3D A549 spheroids established *in vivo* xerographs. In previous studies, PAM suppressed the *in vivo* tumor growth of human ovarian cancer cells [31] and pancreatic cancer cells [24]. Our study also showed significant inhibition of PAM on lung cancer growth; however, the PAM treatment did not suppress *in vivo* tumor growth completely. One possible reason is that the ROS scavenger in living tissues neutralized some amount of ROS generated in PAM. In the future study, combination treatment of PAM with other clinical chemotherapy drugs or radiotherapy should be studied on tumor inhibition.

Mechanically, our previous studies have found that PAM induces cervical cancer cell death by significantly increasing the cleavage of caspase 3 and PARP and activates the MAPK/P38 pathway [32, 33]. The underline mechanisms of PAM treatment in lung cancer 3D spheroids were explored via RNA sequencing analysis. The KEGG enrichment analyses highlighted proliferation- and migration-related pathways, including the MAPK signaling pathway, PI3K/Akt cascade, cell adhesion molecules (CAMs), and cytokine-cytokine receptor interaction, which also suggests that the PAM treatment could affect lung cancer cell function through those downstream pathways.

Western blot analysis showed that after PAM treatment for 48 h, the expression level of phosphorylated PI3K and Akt decreased. This suggests that 20 min-activated PAM inhibits proliferation of 3D A549 spheroids by arresting the PI3K/Akt pathway. A previous study revealed that PAM selectively induced the apoptosis of glioblastoma brain tumor cells through downregulation of p-Akt, a proliferation signal transduction molecular pathway [19]. The present observation of PAM anticancer regulation in 3D spheroids is consistent with 2D cells.

The MAPK pathway regulates changes in cell division, survival, and migration by transmitting extracellular stimuli such as environmental stresses [34]. PAM contains large amounts of ROS and RNS, which generate oxidative stress on cancer cells, thereby frequently activating MAPK signaling in cancer cells [33]. In our study, the activation of MAPK signaling (p38 and ERK MAPK) was observed in 3D lung cancer spheroids upon PAM treatment, indicating the involvement of MAPK signaling in the inhibitory effects of PAM. The combination effect of PAM and ERK inhibitors on cell viability was revealed. Based on our results, we can only conclude that PAM activated MAPKs and downregulate the cell viability and migration of 3D lung cancer spheroids; however, the downstream signal of p-P38 and p-ERK is beyond understanding. And future studies need to be performed to confirm the underlying mechanism. In conclusion, the present study demonstrated the anticancer effect of PAM on the proliferation and metastatic ability of lung cancer cells in 3D spheroids and both *in vitro* and *in vivo* experiments confirmed the antitumor potential of PAM in lung cancer. Moreover, the inhibitory effects of PAM on 3D tumor spheroids resulted from the inhibition of PI3K/Akt and activation of MAPK signaling pathways.

## Figures and Tables

**Table 1:** The primer sequences used for quantitative real-time PCR.

Name	Sequences
Oct4	F 5′-TCCACTTTGTATAGCCGCTGG-3′ R 5′-TGCATACACACAAACACAGCAA-3′
Sox2	F 5′-GGATAAGTACACGCTGCCCG-3′ R 5′-ATGTGCGCGTAACTGTCCAT-3′
Nanog	F 5′-CAATGGTGTGACGCAGGGAT-3′ R 5′-GACTGGATGTTCTGGGTCTGG-3′
Lin-28A	F 5′-ACAATGGGTGGGGGCTATTC-3′ R 5′-GTGTGAACCCAAGCCTGAGA-3′
Slug	F 5′-TGCCTGTCATACCACAACCAGA-3′ R 5′-GGAGGAGGTGTCAGATGGAGGA-3′
Snail	F 5′-AGCCTGGGTGCCCTCAAGAT-3′ R 5′-AGGTTGGAGCGGTCAGCGAA-3′
Twist	F 5′-ACTTCCTCTACCAGGTCCTCCAG-3′ R 5′-CCTCCATCCTCCAGACCGAGAA-3′
ZEB1	F 5′-ACCTGCCAACAGACCAGACAGT-3′ R 5′-ACATCCTGCTTCATCTGCCTGAG-3′
ZEB2	F 5′-CAAGGAGCAGGTAATCGCAAGT-3′ R5′-GCAGTTTGGGCACTCGTAAGGTT-3′
N-Cadherin	F 5′-ACAGTGGCCACCTACAAAGG-3′ R 5′-CCGAGATGGGGTTGATAATGC-3′
E-Cadherin	F 5′-TGCCCAGAAAATGAAAAAGG-3′ R 5′-GTGTATGTGGCAATGCGTTC-3′
18S	F 5′-TCAACTTTCGATGGTAGTCGCCGT-3′ R 5′-TCCTTGGATGTGGTAGCCGTTTCT-3′

## Data Availability

Data are available upon request due to privacy/ethical restrictions.

## References

[1] Motaln H., Recek N., Rogelj B. (2021). Intracellular responses triggered by cold atmospheric plasma and plasma-activated media in cancer cells. *Molecules*.

[2] Yousfi M., Merbahi N., Pathak A., Eichwald O. (2014). Low-temperature plasmas at atmospheric pressure: toward new pharmaceutical treatments in medicine. *Fundamental & Clinical Pharmacology*.

[3] Dai X., Bazaka K., Thompson E. W., Ostrikov K. (2020). Cold atmospheric plasma: a promising controller of cancer cell states. *Cancers*.

[4] Chauvin J., Judée F., Yousfi M., Vicendo P., Merbahi N. (2017). Analysis of reactive oxygen and nitrogen species generated in three liquid media by low temperature helium plasma jet. *Scientific Reports*.

[5] Griseti E., Merbahi N., Golzio M. (2020). Anti-cancer potential of two plasma-activated liquids: implication of long-lived reactive oxygen and nitrogen species. *Cancers*.

[6] Kurake N., Tanaka H., Ishikawa K. (2016). Cell survival of glioblastoma grown in medium containing hydrogen peroxide and/or nitrite, or in plasma-activated medium. *Archives of Biochemistry and Biophysics*.

[7] Judée F., Fongia C., Ducommun B., Yousfi M., Lobjois V., Merbahi N. (2016). Short and long time effects of low temperature plasma activated media on 3D multicellular tumor spheroids. *Scientific Reports*.

[8] Cheung C. H. Y., Juan H. F. (2017). Quantitative proteomics in lung cancer. *Journal of Biomedical Science*.

[9] Nath S., Devi G. R. (2016). Three-dimensional culture systems in cancer research: focus on tumor spheroid model. *Pharmacology & Therapeutics*.

[10] Han K., Pierce S. E., Li A. (2020). CRISPR screens in cancer spheroids identify 3D growth-specific vulnerabilities. *Nature*.

[11] Bielecka Z. F., Maliszewska-Olejniczak K., Safir I. J., Szczylik C., Czarnecka A. M. (2017). Three-dimensional cell culture model utilization in cancer stem cell research. *Biological Reviews of the Cambridge Philosophical Society*.

[12] Ravi M., Paramesh V., Kaviya S. R., Anuradha E., Solomon F. D. (2015). 3D cell culture systems: advantages and applications. *Journal of Cellular Physiology*.

[13] Zhang J., Liu D., Zhang H. (2020). Influence of liquid coverage on the anticancer effects of a helium plasma jet on 3D tumor spheroids. *Plasma Processes and Polymers*.

[14] Plewa J.-M., Yousfi M., Frongia C. (2014). Low-temperature plasma-induced antiproliferative effects on multi-cellular tumor spheroids. *New Journal of Physics*.

[15] Liu P., Zhang R., Yu W. (2017). FGF1 and IGF1-conditioned 3D culture system promoted the amplification and cancer stemness of lung cancer cells. *Biomaterials*.

[16] Li Y., Lv Y., Tang M. (2021). Low-temperature plasma-jet-activated medium inhibited tumorigenesis of lung adenocarcinoma in a 3D in vitro culture model. *Plasma Processes and Polymers*.

[17] Tan A. C. (2020). Targeting the PI3K/Akt/mTOR pathway in non-small cell lung cancer (NSCLC). *Thorac Cancer*.

[18] Kim S. Y., Kim H. J., Kang S. U. (2015). Non-thermal plasma induces AKT degradation through turn-on the MUL1 E3 ligase in head and neck cancer. *Oncotarget*.

[19] Kaushik N. K., Kaushik N., Yoo K. C. (2016). Low doses of PEG-coated gold nanoparticles sensitize solid tumors to cold plasma by blocking the PI3K/AKT-driven signaling axis to suppress cellular transformation by inhibiting growth and EMT. *Biomaterials*.

[20] Wagner E. F., Nebreda A. R. (2009). Signal integration by JNK and p38 MAPK pathways in cancer development. *Nature Reviews. Cancer*.

[21] Urosevic J., Garcia-Albéniz X., Planet E. (2014). Colon cancer cells colonize the lung from established liver metastases through p38 MAPK signalling and PTHLH. *Nature Cell Biology*.

[22] Kang S. U., Cho J. H., Chang J. W. (2014). Nonthermal plasma induces head and neck cancer cell death: the potential involvement of mitogen-activated protein kinase-dependent mitochondrial reactive oxygen species. *Cell Death & Disease*.

[23] Kaushik N., Uddin N., Sim G. B. (2015). Responses of solid tumor cells in DMEM to reactive oxygen species generated by non-thermal plasma and chemically induced ROS systems. *Scientific Reports*.

[24] Azzariti A., Iacobazzi R. M., di Fonte R. (2019). Plasma-activated medium triggers cell death and the presentation of immune activating danger signals in melanoma and pancreatic cancer cells. *Scientific Reports*.

[25] Jo A., Bae J. H., Yoon Y. J. (2022). Plasma-activated medium induces ferroptosis by depleting FSP1 in human lung cancer cells. *Cell Death and Disease*.

[26] Luckert C., Schulz C., Lehmann N. (2017). Comparative analysis of 3D culture methods on human HepG2 cells. *Archives of Toxicology*.

[27] Zhen X., Sun H. N., Liu R., Choi H. S., Lee D. S. (2020). Non-thermal plasma-activated medium induces apoptosis of Aspc1 cells through the ROS-dependent autophagy pathway. *In Vivo*.

[28] Kaushik N. K., Ghimire B., Li Y. (2018). Biological and medical applications of plasma-activated media, water and solutions. *Biological Chemistry*.

[29] Bauer G. (2018). Targeting protective catalase of tumor cells with cold atmospheric plasma- activated medium (PAM). *Anti-Cancer Agents in Medicinal Chemistry*.

[30] Bauer G. (2019). The synergistic effect between hydrogen peroxide and nitrite, two long-lived molecular species from cold atmospheric plasma, triggers tumor cells to induce their own cell death. *Redox Biology*.

[31] Utsumi F., Kajiyama H., Nakamura K. (2013). Effect of indirect nonequilibrium atmospheric pressure plasma on anti-proliferative activity against chronic chemo-resistant ovarian cancer cells in vitro and in vivo. *PLoS One*.

[32] Li Y., Ho Kang M., Sup Uhm H., Joon Lee G., Ha Choi E., Han I. (2017). Effects of atmospheric-pressure non-thermal bio-compatible plasma and plasma activated nitric oxide water on cervical cancer cells. *Scientific Reports*.

[33] Akter M., Jangra A., Choi S. A., Choi E. H., Han I. (2020). Non-thermal atmospheric pressure bio-compatible plasma stimulates apoptosis via p38/MAPK mechanism in U87 malignant glioblastoma. *Cancers*.

[34] Johnson G. L., Lapadat R. (2002). Mitogen-activated protein kinase pathways mediated by ERK, JNK, and p38 protein kinases. *Science*.

